# Mueller matrix analysis of a biologically sourced engineered tissue construct as polarimetric phantom

**DOI:** 10.1117/1.JBO.29.10.106002

**Published:** 2024-10-29

**Authors:** Zixi Lin, Samantha Madnick, Joshua A. Burrow, Jeffrey R. Morgan, Kimani C. Toussaint

**Affiliations:** a Brown University, School of Engineering, Providence, Rhode Island, United States; b Brown University, Department of Pathology and Laboratory Medicine, Providence, Rhode Island, United States; c Brown University, Center for Alternatives to Animals in Testing, Providence, Rhode Island, United States; d Brown-Lifespan Center for Digital Health, Providence, Rhode Island, United States

**Keywords:** polarimetry, Mueller matrix polar decomposition method, engineered tissue, collagen, second-harmonic generation imaging

## Abstract

**Significance:**

The polarimetric properties of biological tissues are often difficult to ascertain independent of their complex structural and organizational features. Conventional polarimetric tissue phantoms have well-characterized optical properties but are overly simplified. We demonstrate that an innovative, biologically sourced, engineered tissue construct better recapitulates the desired structural and polarimetric properties of native collagenous tissues, with the added benefit of potential tunability of the polarimetric response. We bridge the gap between non-biological polarimetric phantoms and native tissues.

**Aim:**

We aim to evaluate a synthesized tissue construct for its effectiveness as a phantom that mimics the polarimetric properties in typical collagenous tissues.

**Approach:**

We use a fibroblast-derived, ring-shaped engineered tissue construct as an innovative tissue phantom for polarimetric imaging. We perform polarimetry measurements and subsequent analysis using the Mueller matrix decomposition and Mueller matrix transformation methods. Scalar polarimetric parameters of the engineered tissue are analyzed at different time points for both a control group and for those treated with the transforming growth factor (TGF)-β1. Second-harmonic generation (SHG) imaging and three-dimensional collagen fiber organization analysis are also applied.

**Results:**

We identify linear retardance and circular depolarization as the parameters that are most sensitive to the tissue culture time and the addition of TGF-β1. Aside from a statistically significant increase over time, the behavior of linear retardance and circular depolarization indicates that the addition of TGF-β1 accelerates the growth of the engineered tissue, which is consistent with expectations. We also find through SHG images that collagen fiber organization becomes more aligned over time but is not susceptible to the addition of TGF-β1.

**Conclusions:**

The engineered tissue construct exhibits changes in polarimetric properties, especially linear retardance and circular depolarization, over culture time and under TGF-β1 treatments. This tissue construct has the potential to act as a controlled modular optical phantom for polarimetric-based methods.

## 1 Introduction

Collagen is the most abundant protein in mammals and is the most prevalent component in the extracellular matrix (ECM).^1^ For collagen type I, which is the most abundant subtype of collagen, the triple helix molecules self-assemble into the macromolecular fibrils, forming a hierarchical structure in various tissues.^2^ This so-called collagen fibrillogenesis process is critical to ECM functions, pathology, cell behaviors, and mechanical properties. Through cell–ECM interactions, collagen architecture also plays an important role in modulating cell behaviors.^3^ To visualize collagen, Masson’s trichrome staining and picrosirius red staining are commonly used together with brightfield microscopy for the histological assessment of connective tissues.^4^^–^^6^ However, to avoid the time-consuming staining process and the associated bias, several label-free imaging technologies are preferred for collagen imaging—taking advantage of its intrinsically highly anisotropic molecular structure. Second-harmonic generation (SHG) microscopy is based on the SHG process, a nonlinear optical process in which two photons interact with a non-centrosymmetric material and produce a beam at exactly half the original wavelength. This quantum mechanical process constrains the effect to a tight focal volume (on the order of femtoliters) for intense optical pulses. When adapted to imaging of biological tissues, the highly selective restriction of this nonlinear process results in images of fibrillar collagen with high specificity and contrast and without any staining.^7^^,^^8^ Several transform-based or gradient-based analysis methods such as Fourier transform, curvelet transform, and edge detection further extract morphological features such as collagen fiber orientation,^9^ alignment,^10^^–^^16^ and diameter.^16^^–^^19^ However, as image processing is needed to calculate these morphological properties from SHG images, polarized light imaging provides an opportunity to directly extract some information about collagen structures with minimum image processing. For example, collagen-fiber orientation has been directly measured and visualized as the fast axis of a wave retarder.^20^

The sensitivity of polarized light to birefringent biological structures and components makes polarization-based imaging a powerful tool for the non-invasive, label-free study of biological samples. Compared with traditional intensity-based imaging techniques, the polarization information extracted from the light–tissue interactions provides valuable additional information about the sample structure and composition.^21^ It is already used to enhance the contrast in the visualization of cancer metastases.^22^ Moreover, as diffusely scattered photons affect the image quality, polarization could also be used to eliminate the unwanted scattering components and hence improve the contrast and resolution in backscattered imaging^23^ and light scattering spectroscopy,^24^^,^^25^ both of which have potential for clinical applications. Although these methods are largely qualitative in their use, the polarimetric properties of the sample could be quantitatively analyzed using the Jones or Mueller formalism. Although the 2×2 complex Jones matrix is limited to represent only non-depolarizing systems, biological samples typically exhibit strong depolarization, which makes the 4×4 real Mueller matrix best-suited for the polarimetric analysis of biological samples.^26^ The Mueller matrix fully describes the polarization properties of a sample which performs a linear transformation of the Stokes vector of an illuminating quasi-monochromatic optical field to an output Stokes vector.^27^ However, the elements of the Mueller matrix are not explicit and independent so the interpretation of each element is not directly relevant to the structure or composition of the sample. Methods such as Mueller matrix polar decomposition (MMPD)^28^^,^^29^ and differential decomposition^30^^,^^31^ enable the extraction of several meaningful scalar polarimetric parameters, which are diattenuation, depolarization, and retardance. Mueller matrix transformation (MMT) provides more rotationally invariant parameters related to the scatterers in the sample.^32^ In addition, compared with SHG microscopy, polarization imaging is not specific to fibrillar collagen. Although in collagen-rich tissues, the birefringence could be considered predominantly originate from fibrillar collagen, many other biological tissue constituents such as elastin,^33^ muscle fibers,^34^ and glucose^35^ also exhibit a polarization response, and it is useful to have a reliable quantitative correlation of the scalar polarimetric parameters with the specific features and composition of the tissues. However, bulk biological tissues comprise complex heterogeneous structures. Thus, it is necessary to have replicative systems that could serve as biological proxies or so-called optical phantoms that are less complex in structure and composition but yet mimic the desired polarimetric properties in typical collagenous tissues and have a controlled polarimetric response.

Tendon tissue is most commonly used to test the polarimetric imaging system because the uniformity of the underlying collagen fibers yields a strong birefringent response.^36^^,^^37^ However, this native tissue lacks standardization in its use as a reference sample for polarimetry because the optical response is susceptible to many factors including the type of animal used for the tissue, the age, and the regional heterogeneity such as fascicles or interfascicular matrix.^38^ In other cases, it has been shown that stretched elastic materials such as polydimethylsiloxane, polycarbonate films, and polyacrylamide can serve as stable and controlled retardation phantoms.^39^^–^^41^ As many of these phantoms are transparent, microspheres or zinc oxide particles are added to mimic the scattering properties of the tissues.^42^^–^^44^ Still, these phantoms generally are too simplified and fail to recapitulate all the optical properties (e.g., reflectivity, scattering, and absorption) of the biological systems of interest, and thus, their utility as a phantom to study the polarimetric response of biological tissues is limited.^45^ Polymerized collagen gels are also used to study the change in polarimetric parameters, especially retardation, as related to collagen density, crosslinking,^46^^,^^47^ and glycation.^48^ Nevertheless, the collagen polymers have less collagen density and also lack the hierarchical structure found in native tissues. Biofabrication methods such as electrospinning have been used to generate anisotropic collagen organization. In these cases, electrospun fiber scaffolds have been used as optical phantoms due to the ability to control fiber diameter and alignment.^49^^,^^50^ The engineered tissue is ultimately formed after the cell seeding process and cell–scaffold interaction,^51^^–^^53^ which modifies its microstructure, and hence, the polarimetric response of the electrospun scaffold is not directly related to the microstructures of the engineered tissues.

To address these limitations, here, we use a biologically sourced engineered tissue construct (BETC) as a novel tissue phantom for polarimetric imaging. These fibroblast-derived, ring-shaped tissue constructs synthesize the highly organized ECM *de novo* and recapitulate not only fibrillar collagen organization but also the mechanical properties of tissues such as the tendon and ligament.^54^ It has been shown that the levels of collagen as well as the strength and stiffness of rings increase as a function of time. We have also confirmed further stimulation of levels of collagen and strength and stiffness of rings by treating with transforming growth factor (TGF)-β1, a master regulator of fibrosis. Here, we investigate the polarimetric response of the tissue construct at different time points and as a function of TGF-β1 treatment during the culture process and identify the most sensitive polarization parameters. We find linear retardance and circular depolarization to be the most sensitive parameters, whereas their respective counterparts, circular retardance and linear depolarization, remain unaffected. This provides an opportunity for the BETC to serve as a controlled and potentially modular optical phantom for polarimetric-based methods. In addition, to further study the potential dependence of the polarimetric properties of the BETC on collagen fiber organization, SHG imaging and accompanying three-dimensional (3D) image analysis are implemented using the approach described in Sec. 2.4. We conclude that both linear retardance and circular depolarization increase across all the time points and that the addition of TGF-β1 accelerates the change of the BETC’s polarimetric properties. The collagen organization of the BETC becomes more aligned over time but is not significantly affected by TGF-β1.

## 2 Method

### 2.1 Sample Preparation

**Fig. 1:**
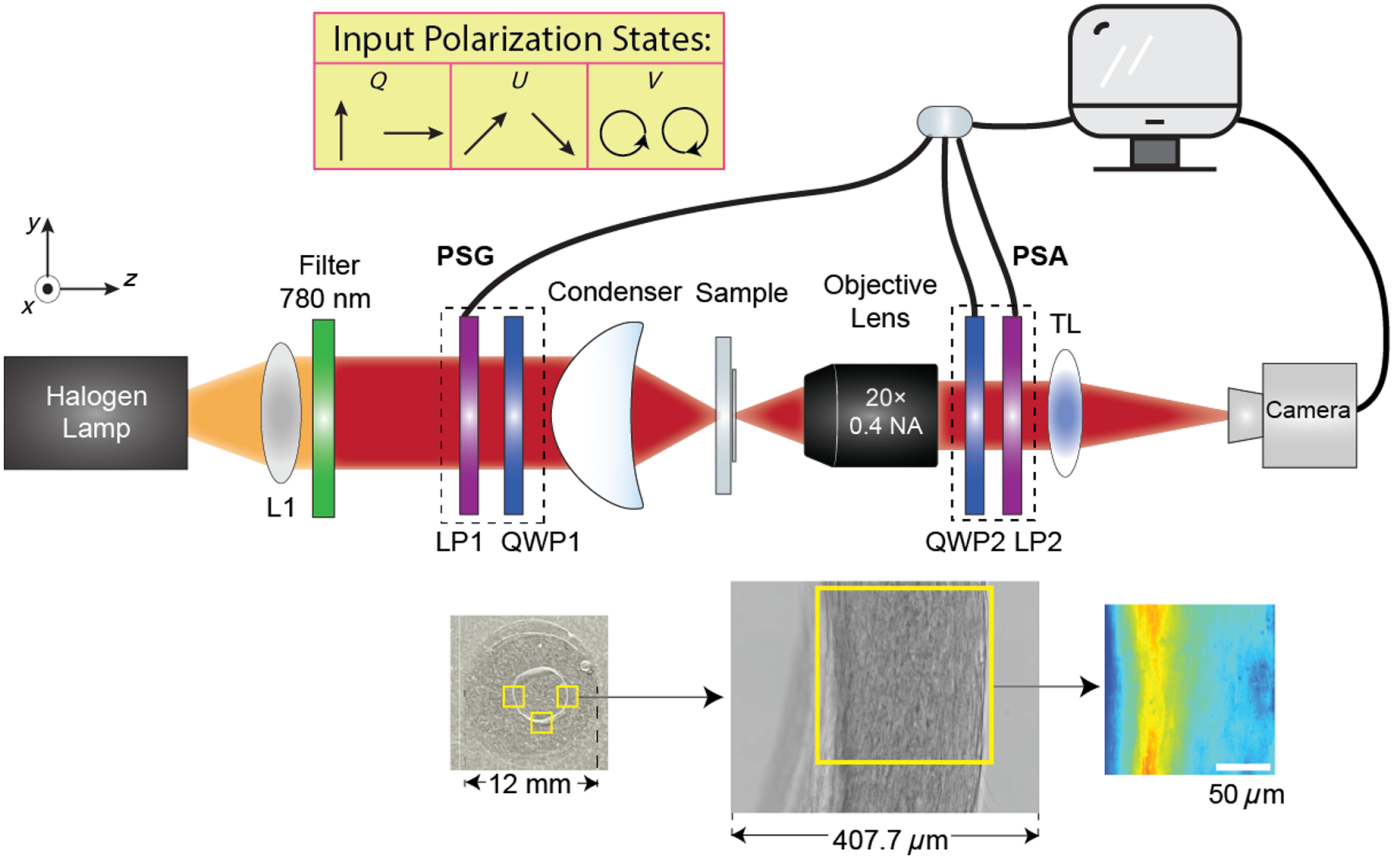
Diagram of the experimental setup for transmission Mueller matrix polarimetry imaging. L, lens; LP, linear polarizer; QWP, zero-order quarter waveplate; TL, tube lens.

The process for forming ring tissues follows a previously published protocol.^54^^,^^55^ The agarose mold (2% agarose) for forming ring tissues, featuring an inner column peg of 5 mm in diameter surrounded by a 0.75-mm-wide cylindrical trough, is formed in standard 24-well plates using a custom-designed, circular, stainless steel mold. Prior to seeding with cells, the agarose molds are equilibrated with a 50:50 mix of serum-free media plus (SFM+) and serum-free medium advanced (SFMA) medium, optimized according to the publication.^54^ The SFM+ medium consists of Dulbecco’s modified Eagle’s medium (DMEM) supplemented with 0.1 mM 2-phospho-L-ascorbic acid and 50.0  μg/mL L-proline. The SFMA medium is advanced DMEM supplemented with 4 mM GlutaMAX. To form ring tissues, each agarose mold is seeded with primary normal human dermal fibroblasts (NHDF) ($3\times 10^{5}\text{ cells}$ per agarose mold) in a 50:50 mix of SFM+/SFMA medium. A concentrated stock of TGF-β1 is reconstituted in 0.1% bovine serum albumin in 4 mM HCl. TGF-β1 is added to 50:50 SFM+/SFMA medium at a concentration of 0 for the control group or 10  ng/ml for the treated group for the duration of the experiment. After adding cells, the 50:50 SFM+/SFMA medium is changed three times per week. During culturing, the thickness of the tissue portion varies, ranging from 170 to 220  μm. At specific time points (days 2, 4, 7, 14, and 21), agarose molds with rings are taken out, washed with phosphate-buffered saline fixed with 10% buffered formalin, and kept at 4°C. Fixed rings are removed from the agarose gel and transferred to glycerol mounting medium in a chambered coverslip and glass microscope slide as shown in the photo in Fig. 1. The rings are not put back after measurements for further culturing.

### 2.2 Optical Setup

Figure 1 represents the Mueller matrix polarimetry setup which is based on a transmitted wide-field imaging system upgraded to a polarization microscope by adding the polarization state generator (PSG) and polarization state analyzer (PSA). The light from the halogen lamp (HL-150-A, AmScope Microscopes, Irvine, California, United States) is collimated by a lens (L1) and then passes through a 780-nm center wavelength spectral filter (FBH780-10, Thorlabs, Newton, New Jersey, United States; FWHM = 10 nm). The PSG lies between L1 and the condenser, consisting of a polarizer for infrared light (LPNIRB100-MP2) and an insertable zero-order quarter-wave plate at 780 nm (WPQ10M-780) subsequently. The light transmitted through the sample is collected by an objective lens (20× magnification with 0.4 NA, Nikon CFI60 TU Plan Epi ELWD), following a tube lens (TL). Between the objective lens and the tube lens, the PSA is placed as the reverse of the PSG, which consists of a zero-order quarter-wave plate following a polarizer. The two polarizers in the PSG and PSA, along with the quarter-wave plate in the PSA, are fixed in motorized rotating mounts and controlled by LabVIEW code. The camera (UI-327xCP-M) captures images with LabVIEW when the PSG and the PSA are rotated to certain configurations. The images are 2056×1542  pixels with an 8-bit image depth. Taking the effective magnification into account, the actual image size is 407.7  μm×305.8  μm as shown in Fig. 1.

To calibrate the polarization components, the null intensity calibration method is first used with the PSG and PSA polarizer oriented with their transmission axes at 90 deg with respect to each other.^27^ A more precise calibration method, the eigenvalue calibration method,^56^^–^^58^ is then applied so that the characteristic matrices, W for the PSG and A for the PSA can be determined pixel-by-pixel with the measurements taken with a set of reference samples such as air, a linear polarizer with axis successively oriented at 0, 45, and 90 deg and a quarter-wave plate with fast axis oriented at 0 and 45 deg.

For a full measurement of the Mueller matrix, a total of 48 intensity images are captured with six distinct input polarization states generated by the PSG and eight configurations of the PSA. The total measurement time for capturing 48 images is less than 2 min. With the use of rolling averaging in this experiment, the total measurement time extends to ∼4 min. The input polarization states, as shown in Fig. 1, include linearly polarized light at $0\;\deg\;(S_{0\;\deg}={\left[1,1,0,0\right]}^{T})$, $45\;\deg\;(S_{45\;\deg}={\left[1,0,1,0\right]}^{T})$, $90\;\deg\;(S_{90\;\deg}={\left[1,-1,0,0\right]}^{T})$, and $-45\;\deg\;(S_{-45\;\deg}={\left[1,0,-1,0\right]}^{T})$ as well as right circularly polarized light ($S_{R}=[1,0,0,1{]}^{T}$), and left circularly polarized light ($S_{L}=[1,0,0,-1{]}^{T}$), where $S_{0\;\deg},S_{45\;\deg},S_{90\;\deg},S_{-45\;\deg}$ are the input Stokes vector when the linear polarizer of the PSG is oriented at 0, 45, 90, and −45 deg; $S_{R}$ and $S_{L}$ are the input Stokes vector when a quarter waveplate is inserted to generate right and left circularly polarized light, respectively, and T, the transpose, indicates that the Stokes vector is a column vector. The PSA configurations perform particular components of the output Stokes vector, including the above input states and two redundant states. The sequence of raw measured intensities is associated with the Mueller matrix (M) of the sample as

B=AMW, (1)

where W is the concatenation of the input Stokes vectors, and A represents how the output Stokes vector is sequentially analyzed by the different configurations of the PSA. The camera measures the intensity of the output Stokes vector passing through eight different PSA configurations sequentially, written as a column vector B in each pixel. In our case, both W and A, with systematic imperfections, are experimentally calculated based on the eigenvalue calibration method and M is uniquely determined pixel-by-pixel based on Eq. (1) with the pseudo-inverse of A and W. The use of 48 images rather than the minimally required 16 images to calculate the Mueller matrix also enhances the robustness and reduces the experimental noise. Additional measurements of a glass slide show an error of ∼1% in derived polar decomposition parameters after eigenvalue calibration.

### 2.3 Calculation of Polarimetric Parameter

Lu–Chipman polar decomposition,^28^^,^^59^ also known as MMPD, is the most widely used mathematical tool to decompose the Mueller matrix into three different fundamental matrices with physical meanings, which describes how the sample changes the polarization state of the incident light. There are three mechanism changes to the state of polarization after interacting with the media, which are diattenuation, retardation, and depolarization. The method thus decomposes an arbitrary Mueller matrix M into the sequential product of three elementary matrices representing a depolarizer ($M_{\Delta }$), a retarder ($M_{R}$), and a diattenuation ($M_{D}$) given by

$$M=M_{\Delta }M_{R}M_{D}.$$ (2)

From the equation, MMPD parameters with physical meaning can further be calculated representing the properties of the sample. The Mueller matrix transformation method is developed to determine the properties of the scatterers in the sample independent of any arbitrary rotation of the sample.^32^ The detailed parameters are listed in Table S1 in the Supplementary Material.

When calculating the average value of the parameters, we use a filtering mask to include only the normal part of the tissue. This mask excludes regions with defects by identifying outliers in depolarization, defined as values exceeding three-scaled median absolute deviations from the median.

### 2.4 Second-Harmonic Generation Imaging and Analysis

We use a multiphoton microscope (Olympus FV-1000-MPE, Olympus, Tokyo, Japan) equipped with a tunable laser (Mai Tai HP, Spectra-Physics, Santa Clara, California, United States) to capture the 3D SHG images of the fixed engineered tissue constructs mounted within the coverslips. The wavelength of the tunable laser is set to 800 nm, and a 405-nm center wavelength filter cube with a 40-nm bandwidth is used to eliminate autofluorescence signals and keep only second harmonic generation signals. We use a 25× (numerical aperture 1.05, water immersion) objective lens to acquire volumetric images (254  μm×254  μm×96  μm). The same regions are chosen despite the relatively smaller field of view of the SHG compared with the polarimetric images. The collagen fiber orientations are estimated from the SHG image stack based on the Fourier transform algorithm developed in MATLAB.^10^^,^^13^ The 3D image stacks are separated into small-volume elements. Within each volume, a local fiber orientation is detected based on the Fourier transform method, represented as (θ,φ), where θ is the in-plane (x-y plane) angle within the image plane and φ is the out-of-plane (z) angle as the angle between the fiber and the image plane. A set of parameters, including the average and standard deviations of the in-plane and out-of-plane angles, and the spherical variance (*SV*) are calculated to quantify the collagen organization of the tissues. The definition of *SV* takes into account the fact that a normalized vector and its reverse vector actually represent the same direction. The *SV* is calculated using the following equation:

$$\mathrm{SV}=1-\frac{\sqrt{{\left(\sum_{i=1}^{n}x_{i}\right)}^{2}+{\left(\sum_{i=1}^{n}y_{i}\right)}^{2}+{\left(\sum_{i=1}^{n}z_{i}\right)}^{2}}}{n},\;0\leq \mathrm{SV}\leq 1,$$ (3)

and then divided by 0.4, which is the maximum *SV* theoretically as already demonstrated in previous publication.^13^ The normalized *SV* ranges from 0 to 1, as 0 implies completely aligned orientations and 1 implies completely disorganized fiber orientations.

### 2.5 Statistical Analysis

Statistical analysis is carried out in JMP (SAS Institute) and R software package (version 4.4.0). Out of the 10 groups, each with five time points consisting of control and TGF-β1–treated groups, eight failed the Shapiro–Wilk test for linear retardance, whereas five failed for circular depolarization. We thus use non-parametric methods throughout the study, as they do not require a normal distribution of the data points. To identify the key parameters related to the time points and treatment groups, Spearman’s correlation is used to test the correlation both among the polarimetric parameters themselves and among the time points, treatment groups, and polarimetric parameters. To determine statistical significance with time points in either control or TGF-β1–treated tissues, the Kruskal–Wallis test with *post hoc* each pair Wilcoxon method is used. We choose to use the original p values rather than adjusted p values because our primary goal is to investigate the changes in polarimetric parameters across different time points so the pairwise comparisons are considered independent from one another. To determine the statistical significance between the control and treatment within each time point, the Wilcoxon test (also called Mann–Whitney U test) is used. The sample size is n=8, 8, 12, 9, and 13 for the control group and n=8, 8, 9, 10, and 9 for the TGF-β1–treated group on days 2, 4, 7, 14, and 21, respectively.

## 3 Results

**Fig. 2:**
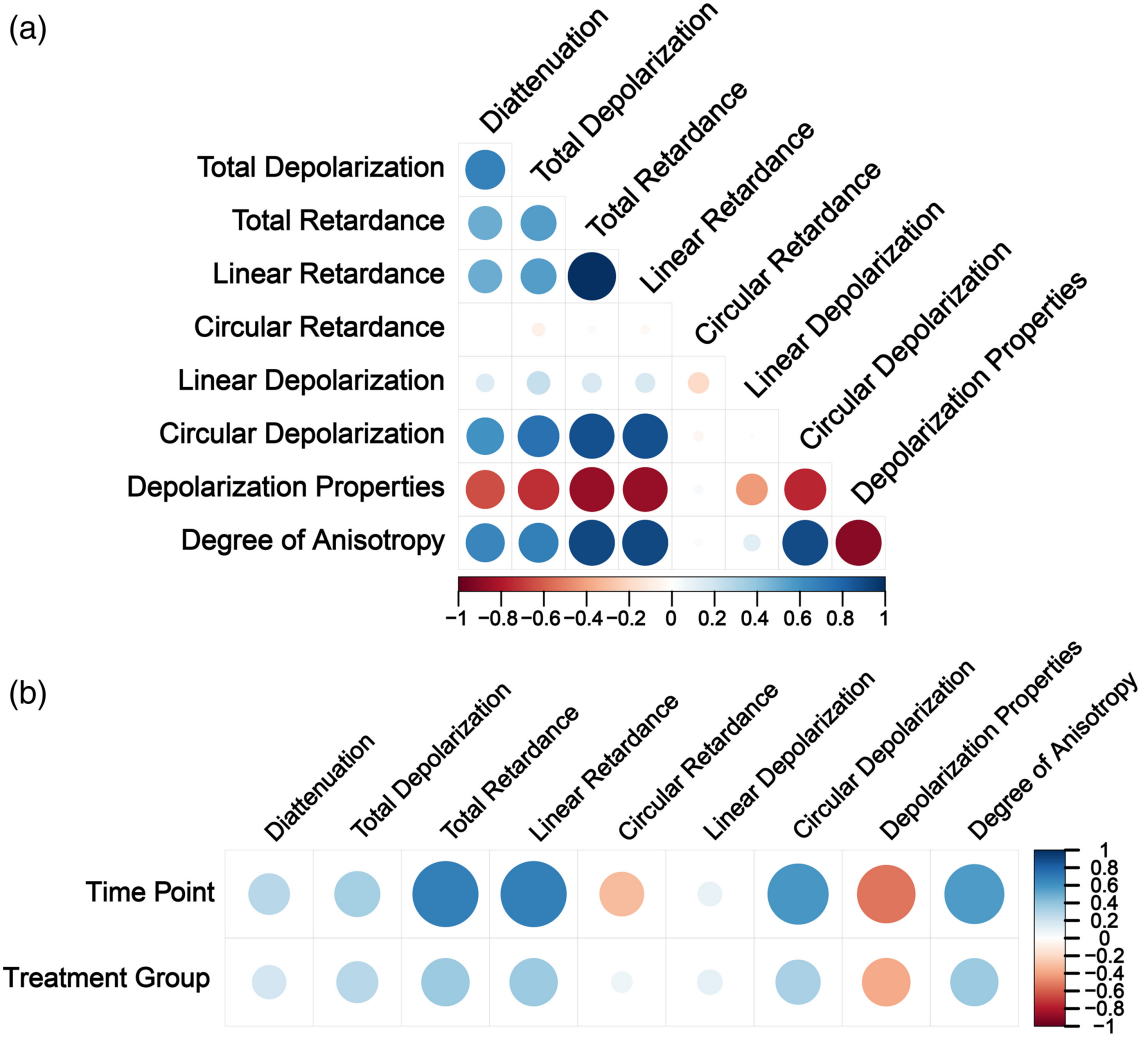
Statistical heatmaps. (a) Matrix of significance circles of the polarimetric parameters. (b) Correlation heatmap of the polarimetric parameters correlated with time points and treatment group.

From the rich bank of polarimetric metrics whose definitions are listed in Table S1 in the Supplementary Material, we first use Spearman’s method to identify the key metrics related to the BETC culturing process. The average values of the polarimetric parameters are used for Spearman’s method. From Fig. 2(a), we find that the circular retardance and linear depolarization have correlation coefficients close to 0 with almost all the other polarimetric parameters. This could be explained by their relatively low values compared with their counter parameters, which are linear retardance and circular depolarization. Table 1 shows the average linear retardance and its corresponding standard deviation for both control tissues and TGF-β1–treated tissues at each time point. Conversely, the averaged circular retardance at all the time points for both the control group and the TGF-β1–treated group is less than 0.35 deg, which is over 10 times smaller than the linear retardance. The fact that the total retardance is linearly correlated with the linear retardance with a correlation coefficient of 1 also indicates that the circular retardance is negligible for the tissue during the culture process. Table 2 shows the average circular depolarization and its corresponding standard deviation for both control tissues and TGF-β1–treated tissues at each time point. On day 4, the control group shows an abnormally high data point for circular depolarization, which contributes to a larger standard deviation. The average linear depolarization ranges from $5.28\times 10^{-3}$ to $9.30\times 10^{-3}$ and are much smaller than the circular depolarization.

**Table 1:** Average linear retardance and its corresponding standard deviation for both control tissues and TGF-β1–treated tissues at each time point.

Linear retardance	Control		TGF-β1 treated	
Time points	Average (deg)	Standard deviation (deg)	Average (deg)	Standard deviation (deg)
Day 2	4.37	0.77	3.73	1.06
Day 4	2.31	0.89	4.00	0.65
Day 7	3.32	1.03	12.55	2.21
Day 14	7.66	1.32	13.23	2.15
Day 21	11.02	2.99	11.96	2.69

**Table 2:** Average circular depolarization and its corresponding standard deviation for both control tissues and TGF-β1–treated tissues at each time point.

Circular depolarization	Control		TGF-β1 treated	
Time points	Average	Standard deviation	Average	Standard deviation
Day 2	$12.83\times 10^{-3}$	$3.04\times 10^{-3}$	$13.25\times 10^{-3}$	$1.934\times 10^{-3}$
Day 4	$22.93\times 10^{-3}$	$28.28\times 10^{-3}$	$14.70\times 10^{-3}$	$3.25\times 10^{-3}$
Day 7	$14.24\times 10^{-3}$	$3.23\times 10^{-3}$	$50.65\times 10^{-3}$	$24.33\times 10^{-3}$
Day 14	$28.47\times 10^{-3}$	$12.55\times 10^{-3}$	$44.39\times 10^{-3}$	$17.42\times 10^{-3}$
Day 21	$35.44\times 10^{-3}$	$12.42\times 10^{-3}$	$43.93\times 10^{-3}$	$15.70\times 10^{-3}$

Spearman’s correlation heatmap in Fig. 2(b) shows that several polarimetric parameters are related to the time points and treatment groups. Based on the previous publication,^54^ the tissue rings continue to grow and mature until day 28. Tissue degradation is not observed within the time range of the study, as it typically occurs after day 28. Given the uneven time points and the inherently nonlinear maturation of engineered tissue, the change in polarimetric properties is positive but not necessarily linear. Therefore, a positive Spearman’s correlation coefficient close to 1 is anticipated for a suitable polarimetric parameter to effectively distinguish among the different time points. The total retardance, linear retardance, circular depolarization, depolarization properties, and degree of anisotropy all show a significant correlation with the time points and treatment groups. The total retardance and linear retardance have almost the same correlation coefficients, which is predictable from the correlation test that the total retardance is linearly correlated with linear retardance. The linear retardance has the highest correlation coefficient with both the time points and treatment groups, which are 0.69 and 0.37, respectively. The circular depolarization has slightly smaller correlation coefficients with the time points and groups, which are 0.59 and 0.32. To closely study the polarimetric response of BETC, we focus on the linear and circular components rather than the total parameter. Specifically, we narrow our focus to two key parameters which are linear retardance (δ) and circular depolarization ($\Delta_{C}$).

**Fig. 3:**
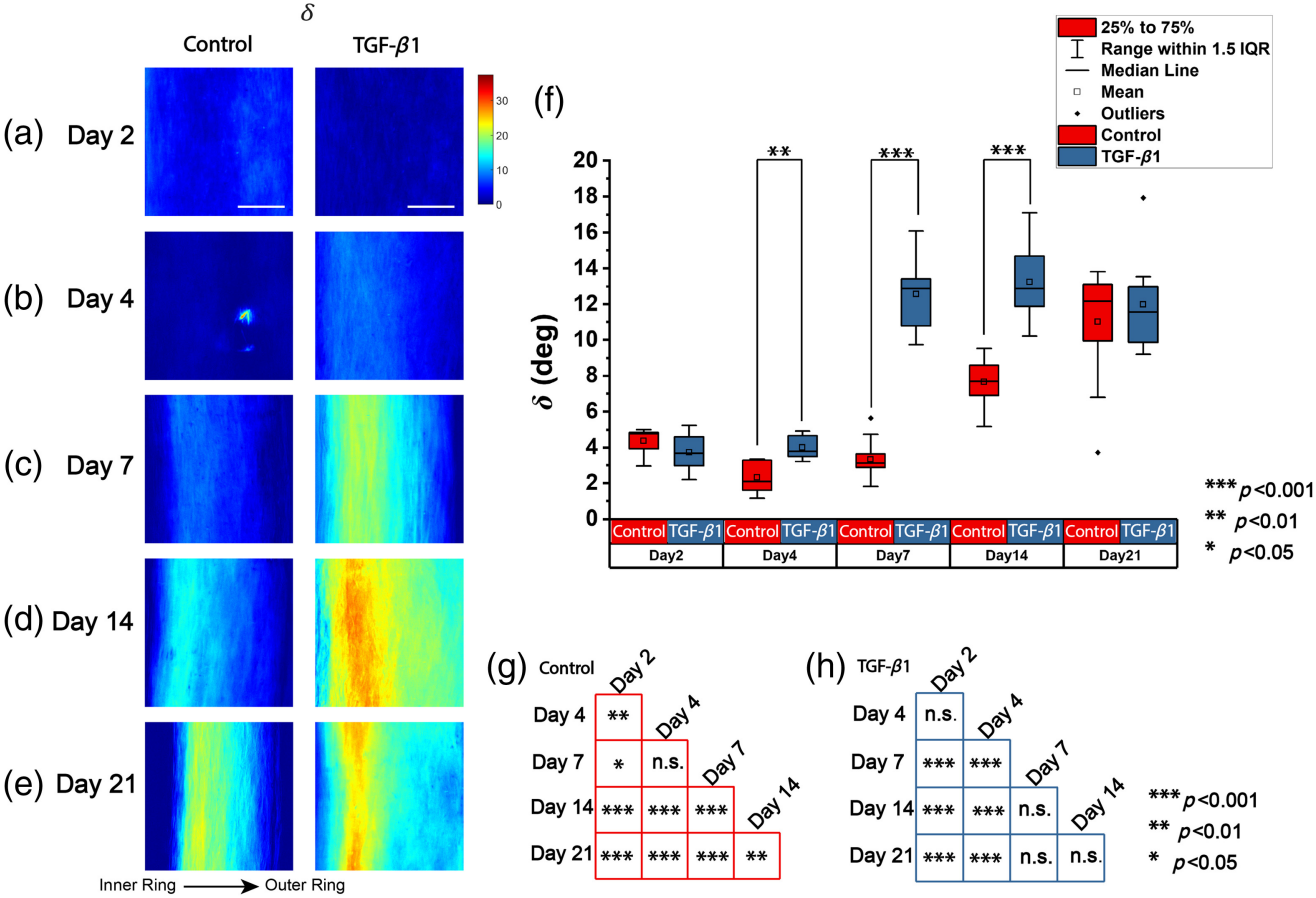
Heatmap of linear retardance of representative ROIs. (a)–(e) Time points of days 2, 4, 7, 14, and 21, respectively. The first column is the control group, and the second column is the TGF-β1–treated group. Scale bar: 50  μm. (f) Boxplot of the average linear retardance. (g) and (h) Significance of difference at different time points within the control group (red) and the TGF-β1–treated group (blue) separately.

Figures 3(a)–3(e) show the representative heatmaps of δ for all the time points and treatments across the regions of interest (ROIs). All the figures are displayed with the same scale bar and color bar. The range of the δ shown here is from 0 to 34.37 deg (which is 0.6 radian), with blue indicating low δ, yellow indicating moderate δ, and red indicating high δ. The first column displays the control group, and the second column displays the treated group. Rows (a) to (e) correspond to specific time points: days 2, 4, 7, 14, and 21 after seeding, respectively. In the control group, despite a small decrease on day 4, δ increases over culture time and changes spatially. In addition, δ is higher for the regions close to the inner ring than to the outer ring.

To best compare the change in δ quantitatively, the average δ in each image is calculated with the whole image, with masked pixels including only the tissue and excluding the part with abnormally high depolarization. The boxplot in Fig. 3(f) is drawn with a solid line indicating the median, a square dot indicating the mean value, the box indicating the middle 50% of all the values, and the whiskers indicating 1.5 times the interquartile range. On average, the treated group has a higher average δ than the control group. The average δ at days 4, 7, and 14 are significantly different with and without TGF-β1 treatment. Both the control and the treated groups have an increase in average δ with culture time (Kruskal–Wallis tests, p<0.001). We then apply the *post hoc* Wilcoxon test to identify which time points are different. The statistical significance of the test is shown in Figs. 3(g) and 3(f) for the control and the TGF-β1–treated groups, respectively. In the control group, the average δ clearly differentiates between each time point. In addition, the average δ at almost all the time points are significantly different from other time points (p<0.05) except for the comparison between days 4 and 7. In the TGF-β1–treated group, the average δ identifies a borderline between days 4 and 7. The average δ before day 7, which refers to the comparison between days 2 and 4, are not significantly different in mean values. The average δ after day 7, which refers to the comparison among days 7, 14, and 21, is not significantly different in the mean values. However, the average δ are significantly different before and after day 7. In addition, the maximum average δ in the TGF-β1–treated group is reached at day 14, with a higher value observed compared with the control group at day 21. Compared with the *post hoc* analysis in the control group which shows that the average δ at day 4 is not significantly different from day 7, we conclude that the addition of TGF-β1 accelerates the increase in δ over time and a significantly higher δ is achieved at an earlier time point.

**Fig. 4:**
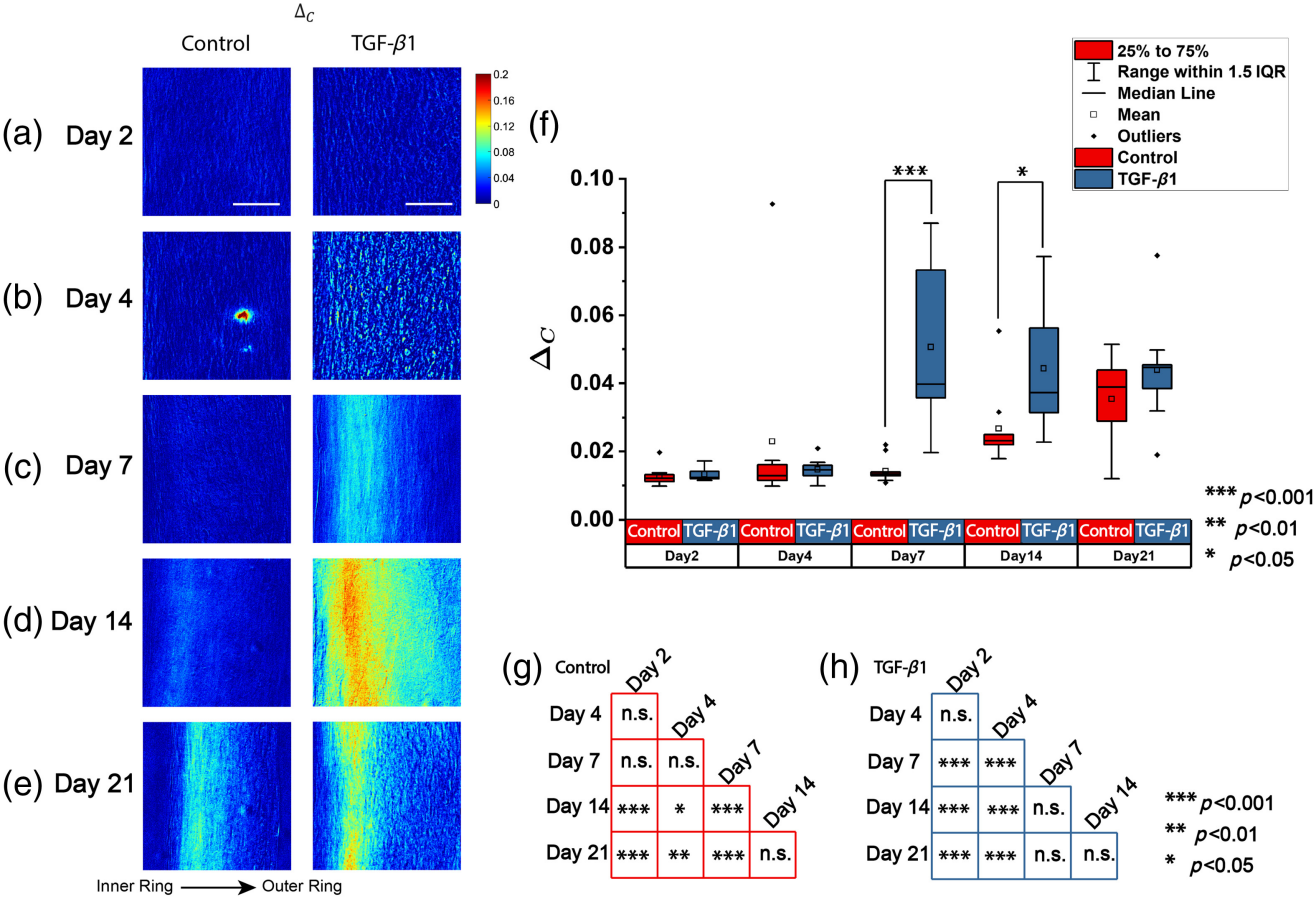
The heatmap of circular depolarization of representative ROIs. (a)–(e) Time points of days 2, 4, 7, 14, and 21, respectively. The first column is the control group, and the second column is the TGF-β1–treated group. Scale bar: 50  μm. (f) Boxplot of the averaged circular depolarization. (g) and (h) Significance of the difference at different time points within the control group (red) and the TGF-β1–treated group (blue) separately.

A similar analysis as above is conducted with circular depolarization. Figures 4(a)–4(e) show the representative heatmaps of $\Delta_{C}$ for all the time points and treatments. All the figures are displayed with the same scale bar and color bar. The range of the $\Delta_{C}$ shown here is from 0 to 0.2, with blue indicating low $\Delta_{C}$, yellow indicating moderate $\Delta_{C}$, and red indicating high $\Delta_{C}$. The first column displays the control group, and the second column displays the treated group. Rows (a) to (e) correspond to specific time points in culture: days 2, 4, 7, 14, and 21 after seeding, respectively. In the control group, the change in $\Delta_{C}$ is not obvious at the earlier time until a slight increase starting at day 14. The change appears to occur close to the inner ring rather than the outer ring. In the TGF-β1–treated group, the change in $\Delta_{C}$ is more notable starting from day 7. Aside from the overall increase starting from day 7, the regions close to the inner ring change more than the outer ring.

After averaging within the ROI, the average $\Delta_{C}$ within each image is summarized and shown in the boxplots in Fig. 4(f). The figure shows a consistent trend as seen in the representative heatmaps comparing the $\Delta_{C}$ between the control and TGF-β1–treated groups. It is shown that both the control and treated groups have an increase in average $\Delta_{C}$ along culture time. The average $\Delta_{C}$ is less sensitive (in its ability to distinguish treatment) at day 4 compared with average δ as the average $\Delta_{C}$ is not significantly different in the control and treated groups. The average $\Delta_{C}$ is still able to distinguish the control and treated groups at day 7 (p<0.001) and day 14 (p<0.05), but it is not as sensitive (in its ability to distinguish at all time points) as average δ in differentiating different cultured time points in the control group either. Although the Kruskal–Wallis tests for either the control group or the treated group justify an increase in the average $\Delta_{C}$ over time (p<0.001), the *post hoc* Wilcoxon tests show that only the borderline is identified by average $\Delta_{C}$. The detailed statistical significance of the *post hoc* Wilcoxon test is shown in Figs. 4(g) and 4(h) for the control and the TGF-β1–treated group, respectively. Compared with the ability of average δ to differentiate all the time points considered in this study, the average $\Delta_{C}$ are similar from days 2 to 7 and also similar from days 14 to 21, but the average $\Delta_{C}$ before and after day 7 are significantly different in value (p<0.05). Similarly, in the TGF-β1–treated group, only a significant increase is recognized between days 4 and 7 (p<0.001), whereas within days 2 to 4 and within days 7 to 21, no significant difference is found separately. The consistency in the specific time points when an abrupt change occurs, as identified by both the average δ and average $\Delta_{C}$, indicates definitive qualitative change in both the structure and composition of the engineered tissue.

The boxplots depicting the change of other polarimetric parameters are shown in Figs. S1(a)–S1(d) and Figs. S2(a)–S2(c) in the Supplementary Material. Figures S1(a) and S1(b) show that circular retardance (Ψ) and linear depolarization ($\Delta_{L}$) are nearly invariant with culture time and treatment, as opposed to the linear retardance and circular depolarization that are very sensitive to the time points and treatment. In addition, the average Ψ is much smaller compared with δ. It results in that the total retardance (R) shown in Fig. S2(c) have the approximately same values δ and are very sensitive in distinguishing the time points and treatment. The average $\Delta_{L}$ is also much smaller than $\Delta_{C}$ but still affect the total depolarization (Δ) shown in Fig. S2(b). The average Δ fails to distinguish the control from the TGF-β1–treated tissues at any time point but does differentiate some time points in the control and TGF-β1-treated groups. Aside from these parameters, the diattenuation (D) is also a fundamental parameter in MMPD. In Fig. S2(a), the average D in the control group only increases slightly from days 2 to 4 but exhibits no significant change after day 4. In the TGF-β1–treated group, the average D increases significantly from days 4 to 7, as consistent with the change in average δ and average $\Delta_{C}$. Although the average D is susceptible to the time points and treatment, the statistical results prove that it is less sensitive compared with the two key parameters, which are δ and $\Delta_{C}$. The two parameters, depolarization properties (b) and degree of anisotropy (A) derived from the MMT method, also exhibit changes related to the culture time and the addition of TGF-β1 treatment as shown in Figs. S1(c) and S1(d). Similar to $\Delta_{C}$, both b and A are able to differentiate the control and treated groups at days 7 and 14. They also identify the same borderline as identified by MMPD parameters, δ and $\Delta_{C}$. However, there is a need for more studies of these parameters in various types of biological tissues to develop a clearer understanding of their quantitative relationship with biological features, and thus, we choose not to focus on these parameters in this work.

**Fig. 5:**
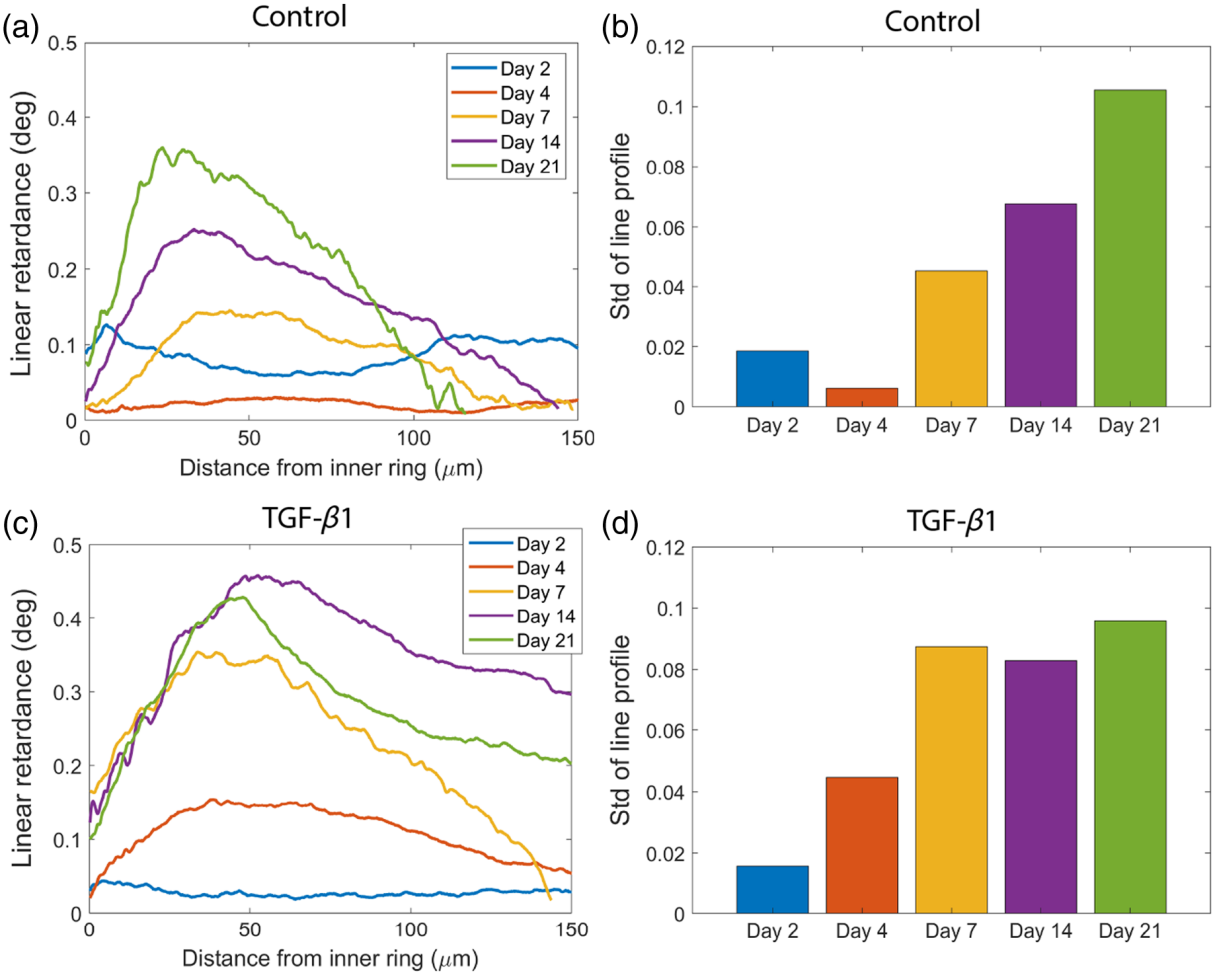
Comparison of linear retardance profiles and standard deviation between control and TGF-β1–treated groups over time. (a) Line profiles of linear retardance for representative ROIs in the control group at each time point, showing variation in retardance across the distance from the inner to the outer ring. (b) Standard deviation of the linear retardance line profiles at each time point for the control group. (c) Line profiles of linear retardance for representative ROIs in the TGF-β1–treated group at each time point. (d) Standard deviation of the linear retardance line profiles at each time point for the TGF-β1–treated group.

Although the boxplots focus on the average parameters over the sample, the polarimetric imaging provides insights into the spatial change of these parameters over cultured time. We crop the line from the representative ROI in the heatmap of δ and extract the line profile of δ along the radial direction from the inner ring to the outer ring. In Fig. 5, the line profiles of δ are displayed as a function of the distance from the inner ring, which is the side attached to the agarose mold during the tissue culture. The lines representing days 2, 4, 7, 14, and 21 are drawn in blue, orange, yellow, purple, and green, respectively. In the control group shown in Fig. 5(a), the line profiles of δ are flat at days 2 and 4, whereas a peak in δ shows up closer to the inner ring rather than the outer ring at day 7 and increases from days 7 to 21. The standard deviation of the line profile is calculated and shown in Fig. 5(b) to demonstrate that the spatial variation of δ continuously increases from days 4 to 21. For the line profile of δ in the TGF-β1–treated group shown in Fig. 5(c), it is shown, from the two graphs with the same limit on the y-axis, that a higher peak of δ is measured than the control group. Although the line on day 2 is still flat, a peak appears on day 4, earlier than observed in the control group. Figure 5(d) shows the standard deviation of the line profile in TGF-β1–treated group. Both the control and TGF-β1–treated groups exhibit a general increasing trend in standard deviation. The most notable differences in standard deviation occur on days 4 and 7, where the treated group’s standard deviations are both 0.04 higher than those in the control group. The percentage increases are 632% on day 4, 93% on day 7, and 23% on day 14. The treated group consistently exhibits greater spatial variation than the control group across days 4, 7, and 14. This further indicates that the addition of TGF-β1 not only accelerates the increase in average δ but also enhances the spatial heterogeneity of δ.

Our results highlight that the BETC exhibits spatial heterogeneity, for which some polarimetric parameters are more sensitive than others. This is expected as many native tissues naturally exhibit spatial heterogeneity. To better show the variation of the engineered tissue polarization properties, we calculate the standard deviations across different ROIs within the same sample. For average δ, two batches exhibit a normalized standard deviation ranging from 1.3% to 18.6%, whereas in another batch, the control samples have greater variation, with a normalized standard deviation ranging from 21.1% to 33%. For average $\Delta_{C}$, more variation is observed, which may be related to the defects in the fabrication process. We further use the Wilcoxon test to show the across-sample variation and show the results in Fig. S2 (see the Supplementary Material). The across-sample variation is affected by the batch with a higher normalized standard deviation. The average δ shows variation across samples at day 2 in the TGF-β1–treated group and on days 4 and 7 in the control group (p<0.05). The average $\Delta_{C}$ shows variation on day 7 in the control group and day 14 in the TGF-β1–treated group (p<0.05). However, biological samples generally show more variation than non-biological phantoms. The average polarimetric parameters may not be the proper parameter to assess sample robustness. The width of the peak and number of pixels effectively used in the calculation would both affect the average polarimetric parameters. In fact, we find that the maximum δ from ROI has excellent robustness both within samples and across samples. Previous studies have also reported the presence of variation in ring thickness, described as non-uniform thin or thick regions. The culture conditions for the tissue construct have already been optimized to produce ring-shaped constructs with improved stability and uniformity,^54^^,^^55^ as demonstrated with a larger sample size than in our study. Further optimization may be feasible to achieve more stable polarization properties, particularly with the spatial distribution.

**Fig. 6:**
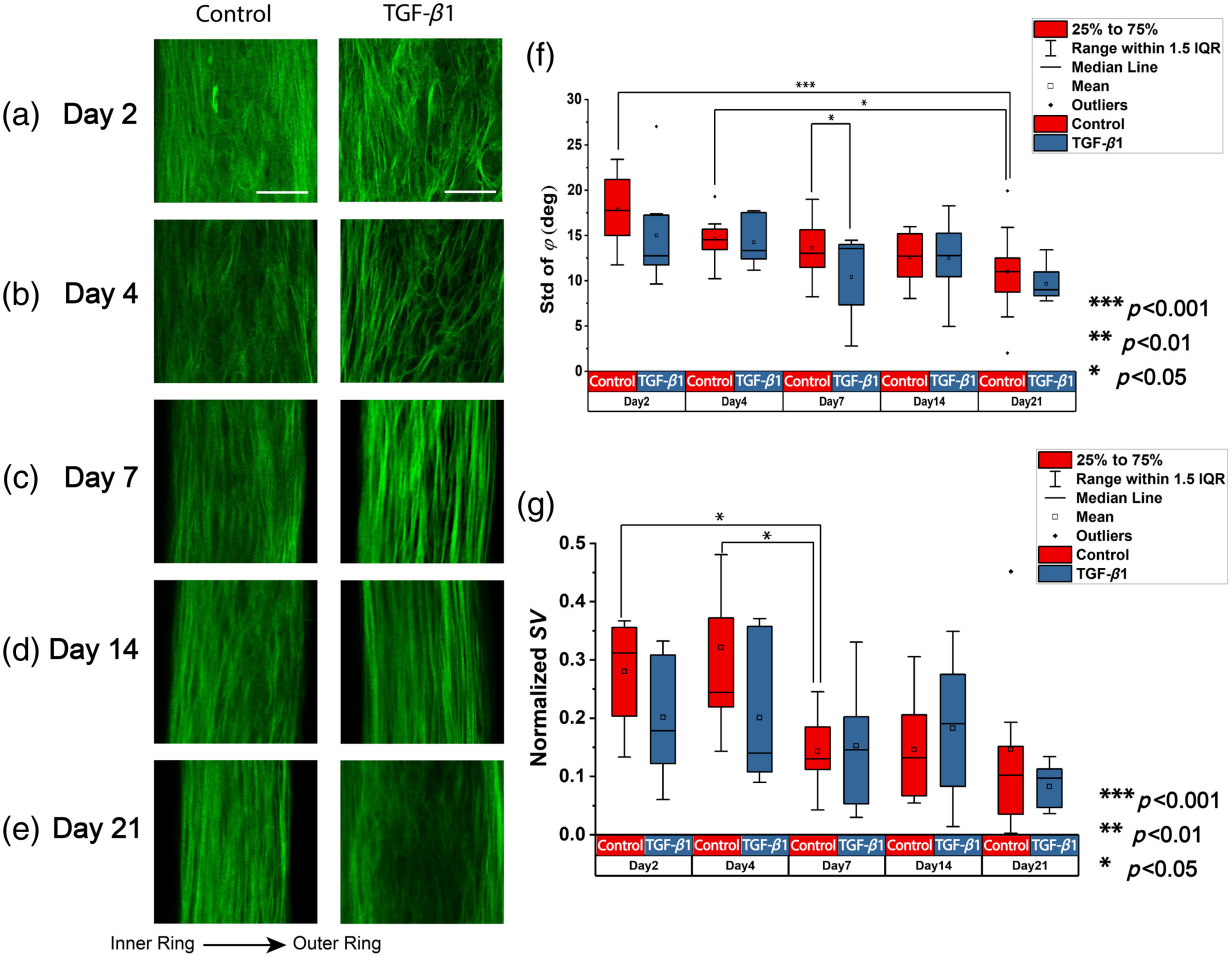
Selective 2D representative ROIs from second-harmonic generation image stacks. (a)–(e) Time points of days 2, 4, 7, 14, and 21, respectively. The first column is the control group, and the second column is the TGF-β1–treated group. Scale bar: 50  μm. (f) Boxplot of the out-of-plane angle standard deviation. (g) Boxplot of the normalized spherical variance of the 3D orientations.

To explore the potential dependence of the polarimetric properties on the spatial organization of collagen fibers during BETC culturing, 3D SHG image stacks are used to study the collagen structural change. Figures 6(a)–6(e) show the representative two-dimensional (2D) image slices of all the time points and treatments. The representative 2D image slices are cropped with the same ROI as with the polarimetric images, but the 3D orientation analysis is conducted with the whole image. As a result of the 3D sectioning in SHG imaging, the ring sizes seen in SHG images are not the same as in the polarimetric images. The boxplot in Fig. 6(f) shows the standard deviation of the out-of-plane angle (φ) at different time points and treatments. The out-of-plane angle refers to the orientation toward the imaging plane and the standard deviation of the out-of-plane angles quantifies the collagen fiber alignment relative to the imaging plane. The boxplot in Fig. 6(g) shows the 3D spherical variance of the 3D collagen fiber orientations at different time points and treatments, quantifying the alignment of the collagen structure in 3D. Both the normalized *SV* and the standard deviation of φ have a trend of decreasing over time in the control and treated groups (p<0.01), indicating the collagen structure turns out to be more aligned as the tissues grow from days 2 to 21. However, the two parameters are not able to distinguish between the control and TGF-β1–treated groups at almost all the time points.

## 4 Discussion

Our study shows that the ring-shaped BETC exhibits changes in polarimetric properties over time and under TGF-β1 treatment. The increase in the linear retardance and circular depolarization are significant and can serve as quantitative metrics to assess the engineered tissue culture process non-invasively and effectively. The fact that the change in linear retardance and circular depolarization can be controlled independently of their counterparts, circular retardance, and linear depolarization, indicating that the BETC can serve as controlled polarimetric phantoms that mimic biological tissue properties.

Currently, although the polarimetric parameters of biological tissues are not necessarily independent from each other, existing polarimetric phantoms are designed to focus mostly on a single parameter, e.g., linear retardance.^45^ Unfortunately, it remains a challenge for these existing phantoms to recapitulate the polarization response of biological tissues, which, in general, are integrated. For the tissue construct used in this study, the *de novo* tissue culture process better mimics the optical properties of native tissue as it enables the simultaneous change in linear retardance, circular depolarization, and diattenuation, independent from circular retardance and linear depolarization. We observe that the BETC yields much higher circular depolarization than linear depolarization, just as reported in native tendon tissues,^60^ which is in contrast to traditional phantoms relying on the use of microspheres.^61^ Although the change in linear retardance is well-known to be closely related to birefringence, primarily arising from collagen, the circular depolarization properties are not clearly known in biological tissues. The polarization dependence of scattering and penetration depth is influenced by local variations in optical path length across different tissue types.^60^ The transition from locally wavy fibrils to straight fibrils within a given volume can potentially increase the portion of circularly polarized light converted into linear polarized or unpolarized light. We note that the BETC has values of depolarization less than 0.1, which is much smaller than native tissues that generally exhibit strong depolarization greater than 0.8 to 0.9.^62^ We owe the significant difference to the glycerol mounting used for optical clearing.^44^^,^^63^^,^^64^ In addition, the linear retardance in BETC is likely in the order of $10^{-4}$ for an average thickness of ∼240  μm, which is much smaller than in native tendon tissues ($∼10^{-3}$).^39^^,^^65^ However, the value of linear retardance for BETC is on par with what has been obtained for other optical phantoms.^39^

In terms of spatial organization, metrics such as normalized SV and standard deviation of φ prove that the tissue gradually becomes more similarly organized over time for both the control and TGF-β1–treated groups, thereby indicating that TGF-β1 does not affect collagen fiber spatial organization. It is consistent with our understanding that TGF-β1 accelerates the deposition of collagen. The quantitative metrics representing the spatial organization fail to distinguish among different time points, suggesting that collagen alignment is not the major cause of the change in polarimetric response during the collagen synthesis process shown in the fabrication of BETC. Previous studies have indicated that the change in collagen content is more significant^54^ and is potentially a more important factor affecting the polarimetric properties of the tissues.^47^ However, the lack of observed differences with spatial organization could also potentially be attributed to the presence of the mold for the BETC, as the mold constraints and guides the cell alignment.^66^

The 3D ring tissues analyzed in this paper synthesize a collagen-rich fibrous extracellular matrix *de novo*, making them a promising *in vitro* model for studying fibrosis—a major unmet medical need. It is a complete cell-based model that relies on cell-mediated tension to direct anisotropic cellular alignment and collagen synthesis. The BETC fabrication process can be further fine-tuned in a controlled manner with varying dosages of TGF-β1,^54^ offering a straightforward approach to study the polarimetric response during collagen synthesis. Treatment with drugs such as PAT-1251 (an inhibitor of collagen crosslinking), CZ415 (an inhibitor of collagen synthesis), or cytokines such as interleukin-13 and tumor necrosis factor alpha is also promising to alter the polarization properties of the BETC. Moreover, changing the shape of the agarose mold or using different cell types could induce regional-specific collagen fiber organization, enabling the phantom to mimic a broader range of tissues beyond the tendon.^66^ In addition, unlike studies that focus on simple processes such as collagenase-induced damage or dehydration using polarization imaging,^67^^,^^68^ the collagen synthesis process we studied and perturbed has greater biological significance. When coupled with the polarimetric measurements, the tissue construct as an alternative to animal testing can be used to determine which growth factors and cytokines drive fibrosis as well as screen for drugs that may be potential therapies.

## 5 Conclusion

In this study, we use a customized polarization microscope to measure the polarimetric properties of the engineered tissue over culture time and with different treatments. MMPD and MMT are used to calculate meaningful scaler polarimetric parameters from the Mueller matrix pixel by pixel. We further identify two key parameters that are most sensitive to all the time points and treatments, which are linear retardance and circular depolarization. Using statistical methods, we determine that both linear retardance and circular depolarization increase across all the time points. Specifically, the linear retardance could be distinguished across all the time points in the control group, whereas in the treated group, only a significant increase was found from days 4 to 7. The circular depolarization shows an increase from days 7 to 14 in the control group, whereas in the TGF-β1–treated group, the increase happens earlier from days 4 to 7. We conclude that the addition of TGF-β1 not only accelerates the growth of engineered tissue but also increases the maximum linear retardance that the tissue could achieve, showing more potential methods to tune the polarimetric properties of the BETC. Conversely, the counterparts of the key polarimetric parameters, which are circular retardance and linear depolarization, are insusceptible to culture time and treatment. This offers the potential for the BETC to act as a controlled and potentially modular optical phantom for polarimetric-based methods. Moreover, SHG imaging is taken following the 3D orientation analysis based on the Fourier transform to quantify the collagen–fiber organization. We determine that the collagen-fiber organization of the BETC becomes more organized over time but is not affected by TGF-β1 treatment. The ring-shaped, BETC bridges the gap between the non-biological optical phantoms and native biological tissues as the engineered tissue mimics certain polarimetric properties of tissues. In addition, a realistic tissue phantom with tunable polarimetric properties could facilitate the development of optical physiological sensors. For further study, the ring-shaped BETC could be treated with other drugs and cytokines known to affect collagen synthesis and collagen crosslinking and tested for their effects on the polarimetric properties.^54^

## Supplementary Material



## Data Availability

All data needed to evaluate the conclusions in the paper are present in the paper and/or the Supplementary Material. Additional data related to this paper may be requested from the authors.
