# Supplementary material for: Mueller matrix analysis of a biologically sourced engineered tissue construct as polarimetric phantom
Source: J Biomed Opt. 2024 Oct 29;29(10):106002. doi: 10.1117/1.JBO.29.10.106002 (PMC11521148; doi:10.1117/1.JBO.29.10.106002)
Supplement: Supplementary file 1 [file JBO_029_106002_SD001.pdf]

Supplementary Material

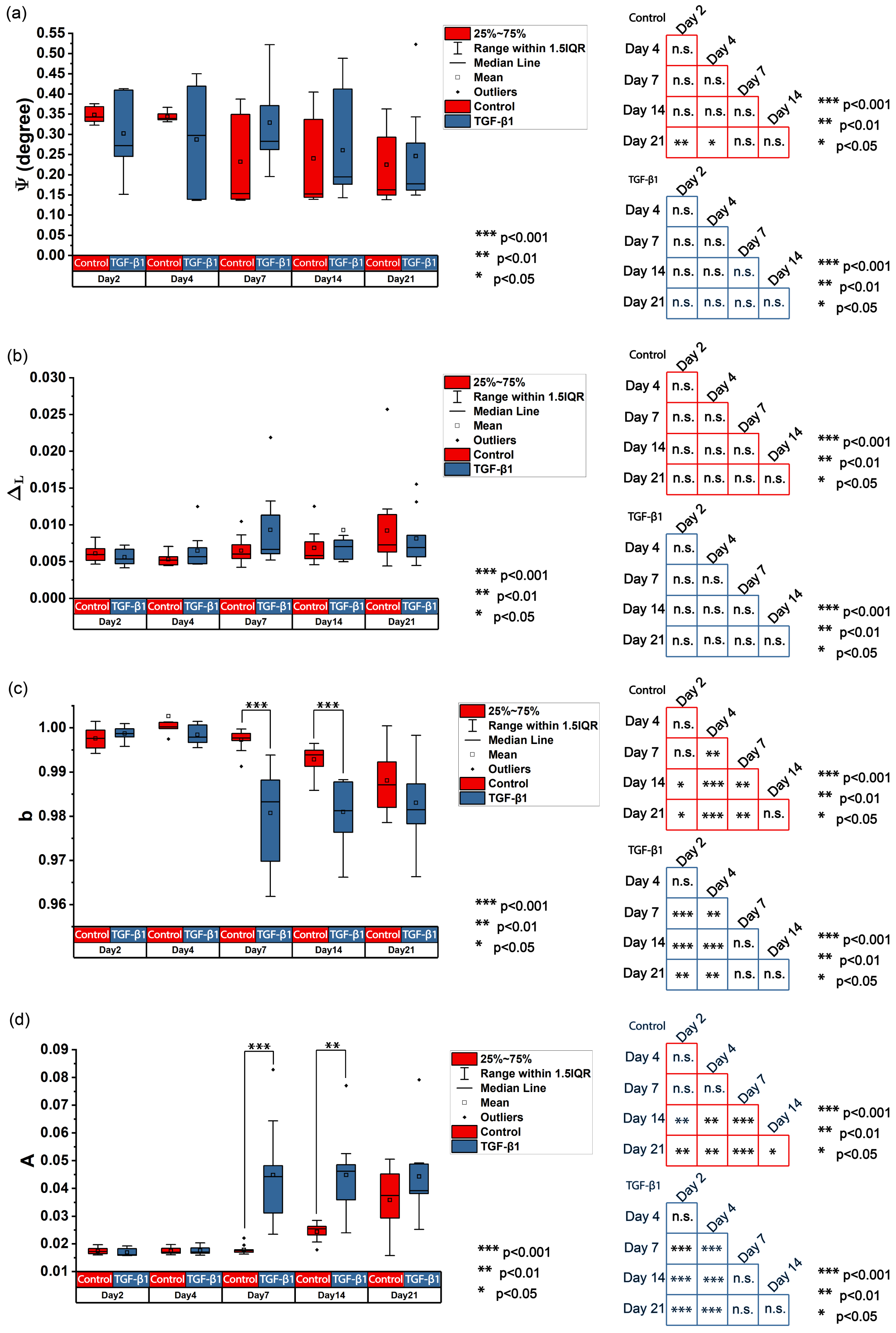

Fig. S1 Boxplot of the averaged polarimetric parameters (a) circular retardance, (b) linear depolarization, (c) depolarization properties (d) degree of anisotropy and the significance of difference at different time points within the control group (red) and TGF-β1 treated group (blue), separately.

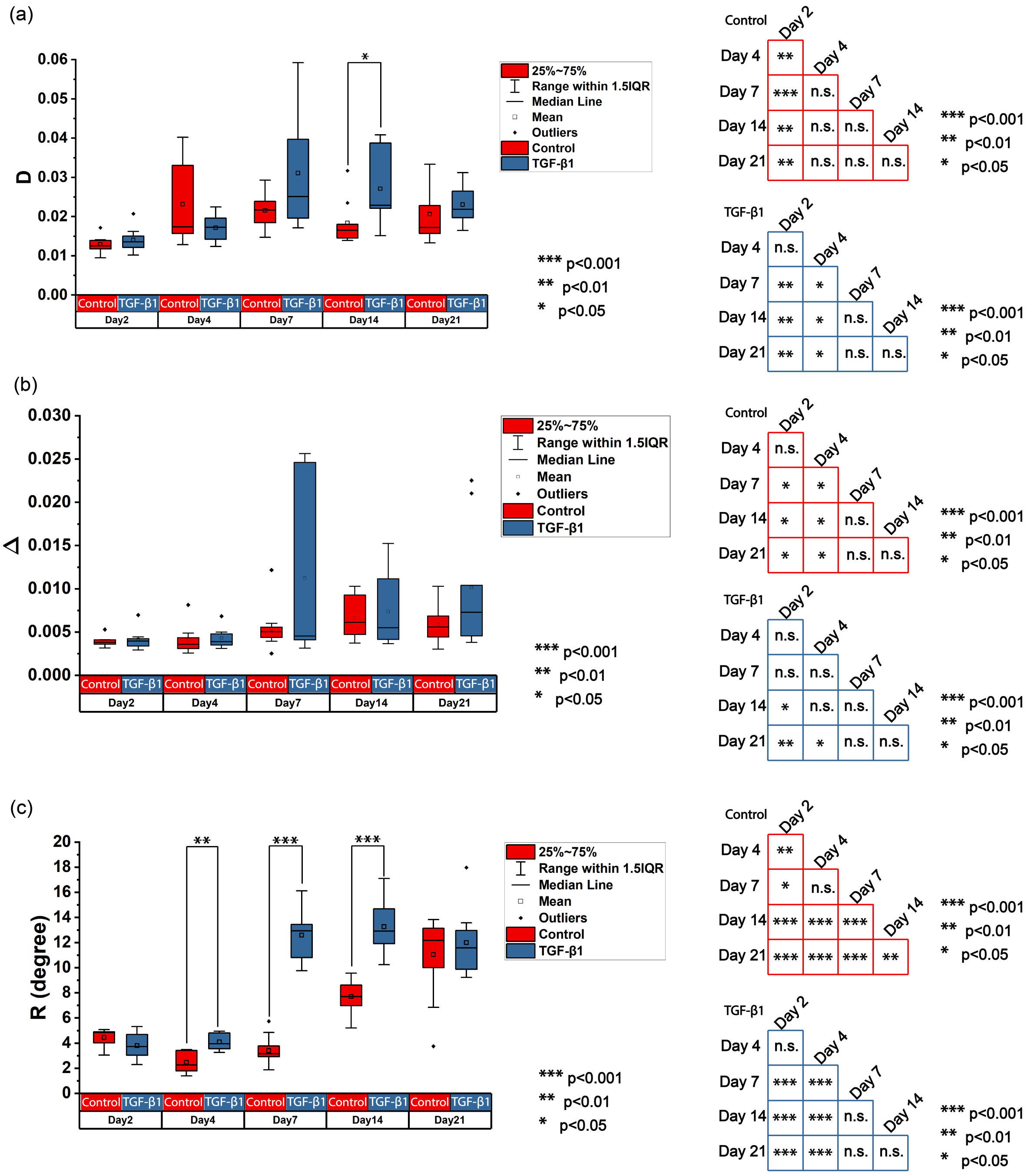

Fig. S2 Boxplot of the averaged polarimetric parameters (a) diattenuation, (b) total depolarization, (c) total retardance and the significance of difference at different time points within the control group (red) and TGF-β1 treated group (blue), separately.

(a) δ

	Control	TGF-β1
Day 2	n.s.	*
Day 4	*	n.s.
Day 7	*	n.s.
Day 14	n.s.	n.s.
Day 21	n.s.	n.s.

(b) Δ_C

	Control	TGF-β1
Day 2	n.s.	n.s.
Day 4	n.s.	n.s.
Day 7	*	n.s.
Day 14	n.s.	*
Day 21	n.s.	n.s.

*** $p<0.001$
** $p<0.01$
* $p<0.05$

Fig. S3 Statistical comparisons showing the across sample variations of (a) linear retardance (δ) and (b) circular depolarization (Δ_C) at each time point (days 2, 4, 7, 14, and 21) for both the control and TGF-β1 treated groups.

Table S1 List of scalar polarimetric parameters derived using MMPD and MMT.

Method	Parameter	Equation
MMPD	Diattenuation	$D = norm(\vec{D}) = \sqrt{\boldsymbol{M}(1,2)^2 + \boldsymbol{M}(1,3)^2 + \boldsymbol{M}(1,4)^2}$ $0 \leq D \leq 1$
	Total Depolarization	$\Delta = 1 - \frac{\lvert tr(\boldsymbol{M}_\Delta) - 1\rvert}{3}$ $0 \leq \Delta \leq 1$
	Total Retardance	$R = cos^{-1}\left[\frac{tr(\boldsymbol{M_R})}{2} - 1\right]$ $0 \leq R \leq \pi$

	Linear Retardance	$\delta = cos^{-1}\{\sqrt{[\boldsymbol{M_R}(2,2) + \boldsymbol{M_R}(3,3)]^2 + [\boldsymbol{M_R}(3,2) - \boldsymbol{M_R}(2,3)]^2} - 1\}$ $0 \leq \delta \leq \pi$
	Circular Retardance	$\Psi = tan^{-1}\left(\frac{\boldsymbol{M}_R(3,2) - \boldsymbol{M}_R(2,3)}{\boldsymbol{M}_R(2,2) + \boldsymbol{M}_R(3,3)}\right)$ $0 \leq \Psi \leq \pi$
	Linear Depolarization	$\Delta_L = 1 - \frac{\boldsymbol{M}(2,1) + \boldsymbol{M}(2,2)}{\boldsymbol{M}(1,1) + \boldsymbol{M}(1,2)}$ $0 \leq \Delta_L \leq 1$
	Circular Depolarization	$\Delta_C = 1 - \frac{\boldsymbol{M}(4,1) + \boldsymbol{M}(4,4)}{\boldsymbol{M}(1,1) + \boldsymbol{M}(1,4)}$ $0 \leq \Delta_C \leq 1$
MMT	Depolarization Properties	$b = \frac{\boldsymbol{M}(2,2) + \boldsymbol{M}(3,3)}{2}$
	Degree of Anisotropy	$A = \frac{2bt}{b^2+t^2}$, where $t = \frac{\sqrt{(\boldsymbol{M}(2,2) - \boldsymbol{M}(3,3))^2 + (\boldsymbol{M}(2,3) + \boldsymbol{M}(3,2))^2}}{2}$
